# Chemical and Bioactive Features of *Amaranthus caudatus* L. Flowers and Optimized Ultrasound-Assisted Extraction of Betalains

**DOI:** 10.3390/foods10040779

**Published:** 2021-04-05

**Authors:** Custódio Lobo Roriz, Virginie Xavier, Sandrina A. Heleno, José Pinela, Maria Inês Dias, Ricardo C. Calhelha, Patricia Morales, Isabel C. F. R. Ferreira, Lillian Barros

**Affiliations:** 1 Centro de Investigação de Montanha (CIMO), Instituto Politécnico de Bragança, Campus de Santa Apolónia, 5300-253 Bragança, Portugal; cmlobo@ipb.pt (C.L.R.); virginie.xavier@ipb.pt (V.X.); jpinela@ipb.pt (J.P.); maria.ines@ipb.pt (M.I.D.); calhelha@ipb.pt (R.C.C.); iferreira@ipb.pt (I.C.F.R.F.); 2 Departamento de Nutrición y Ciencia de los Alimentos, Facultad de Farmacia, Universidad Complutense de Madrid (UCM), Pza Ramón y Cajal s/n, E-28040 Madrid, Spain; patmoral@ucm.es

**Keywords:** *Amaranthus caudatus* L., bioactive compounds, betacyanins, natural colorants, extraction process optimisation

## Abstract

The vibrant colours of many plants are due to secondary metabolites, such as nitrogen-containing compounds, where betacyanins are included. These compounds can be found in plants such as *Amaranthus caudatus* L. that, due to their high nutritional benefits, have been overproduced, which leads to the accumulation of large amounts of bio-residues. Among these bio-residues, the flowers which have a very intense pink colour and present no economic value or subsequent destination can be exploited as sources of natural colouring agents (betacyanins). This work aimed at characterising the flower’s extract in terms of bioactive molecules such as tocopherols, organic acids, but essentially in terms of betacyanins, in order to obtain a natural colouring agent. For the extraction, ultrasound-assisted extraction (UAE) ideal conditions were obtained using the Response Surface Methodology (RSM), allowing the attainment of an enriched extract of betacyanins in high yields and purity. The obtained extracts were analysed for their bioactive potential, namely antioxidant, antimicrobial and cytotoxic properties. From the obtained results, three isoforms of tocopherols were detected, β-tocopherol (0.884 ± 0.003 mg/100 g dry weight (dw)) being the most abundant one. Regarding the organic acids, oxalic (2.48 ± 0.05 mg/100 g dw), shikimic (0.170 ± 0.003 mg/100 g dw) and traces of fumaric acid were found. Four betacyanins were identified and quantified, namely: amaranthine (171 ± 1 mg/g extract), isoamaranthine (38 ± 1 mg/g extract), betanin (1.6 ± 0.1 mg/g), and isobetanin (1.3 ± 0.1 mg/g extract). The obtained extract also presented antioxidant activity with inhibition concentration (IC_50_ values) of 29.0 ± 0.4 μg/mL and 114 ± 4 μg/mL for Δ*t* of 60 min and 120 min, respectively in the oxidative haemolysis inhibition assay (OxHLIA) assay. The obtained extract also presented an interesting antibacterial activity with minimum inhibitory concentrations ranging from 5 to 20 mg/mL against pathogenic bacteria and revealed no toxicity for normal cells.

## 1. Introduction

Secondary metabolites in the plant kingdom are natural products resulting from the primary metabolism responsible for various physiological functions. These molecules are accountable for the strong interaction of plants with the surrounding environment, ensuring their survival [1]. Plants have many different strategies to survive and ensure their propagation, such as attractive odours, or vibrant colours in their flowers and fruits. These vibrant colours result from the expression of some molecules from secondary metabolism, that act as a gimmick, attracting different animals responsible for pollinating them [2].

One of the most important classes of molecules present in plants is nitrogen-containing compounds, including betalains, that are alkaloids responsible for the intense colours of several flowers and fruits of the Caryophyllales [3,4]. Unlike anthocyanins, betalains are not dispersed by a huge range of plant species [4], but they can be found in different plant tissues of flowers, fruits, stems, leaves and even in seeds [5]. Betacyanins, a more specific subgroup of betalains, are responsible for the bright pink colour of the plants, aiming at attracting different animals as pollinating or seed dispersing agents [6]. These secondary metabolites have been extensively explored, due to the exhaustive search of the industry for natural colouring agents, caused by the recent limitations imposed by legislators regarding the side effects of artificial compounds and also due to the consumer´s demand for safer and healthier products [7].

*Amaranthus caudatus* L. has gained special attention in recent years due to the nutritional profile of its seeds [8], and also to numerous bioactivities responsible for the therapeutic potential of this species [9]. The significant increase in the production of this plant led to the accumulation of high amounts of by-products, such as the pink flowers that have no valuable destination. These flowers present a strong pink colour that can be exploited as sources of natural colouring agents (betacyanins) for industrial application, giving an economically viable destination for these by-products. In addition to the interesting colouring power presented by betacyanins, this compounds can be associated with many biological properties such as antioxidant, hypoglycaemic and hypolipidemic action, antimicrobial activity [10], which make the obtained colouring extract, safer, healthier and functional [11]. In the literature it is possible to witness the growing amount of information regarding these compounds and their extraction methodologies [12]. Some examples of alternative sources of these compounds are the flowers of *Gomphrena globosa* [12], and *Bougainvillea glabra* [13], as well as fruits of *Hylocereus* spp. [14]. Their extraction can be carried out by numerous methodologies and, for instance, it is possible to find in the literature authors exploring the recovery of these compounds with extraction by maceration [15], microwave-assisted extraction [16] and ultrasound-assisted extraction [14,16], this last one being very interesting and showing the most promising results.

Bearing this in mind, the present work aims at exploiting the flowers of *A. caudatus* as a source of bioactive (tocopherols and organic acids), but specially of colouring compounds (betacyanins).

Ultrasound assisted extraction (UAE) was applied and optimised through the Response surface methodology (RSM), a statistical tool widely used in the optimisation of extraction processes, which allows the evaluation of several factors that affect the extractability and stability of the target molecules and already applied to the UAE technique, in order to recover colouring agents from the same family [16]. Moreover, the bioactive potential of these extracts will also be evaluated through the antioxidant and antimicrobial potential, as well as the hepatotoxicity for normal cells.

## 2. Materials and Methods

### 2.1. Plant Samples

Samples of *Amaranthus caudatus* L. were supplied by the company “Cantinho das Aromáticas” (Portugal), an enterprise located in Vila Nova de Gaia, Portugal that commercialises aromatic and medicinal plants. This company has a vast certified collection of plant materials of local and exotic origin and applies sustainable organic farming practices. The botanical identification was made by the botanical specialist responsible for the collection of medicinal plants in the herbarium of Escola Superior Agrária (BRESA) of the Polytechnic Institute of Bragança, Portugal. The *A. caudatus* flowers were manually separated from the rest of the plant, lyophilised (FreeZone 4.5, Labconco, Kansas City, MO, USA), grounded to 20 mesh, and kept in a container protected from light and humidity at −20 °C until further analysis.

### 2.2. Colour Measurement

The colour of the lyophilised flower sample was measured with a colorimeter (model CR-400, Konica Minolta Sensig Inc., Tokyo, Japan) previously calibrated against a standard white tile, equipped with an iluminant C and an 8-mm diaphragm aperture. The CIE L* (lightness), a* (greenness–redness), and b* (blueness–yellowness) colour space values were recorded using a Spectra Magic Nx software (version CM-S100W 2.03.0006)

### 2.3. Chemical Characterisation

#### 2.3.1. Organic Acids

For organic acids extraction, 1 g of flower samples was extracted with 25 mL of metaphosphoric acid at 4.5% solution, that was then filtered (0.22-µm disposable filters), for further analysis. Organic acids were analysed by ultra-fast liquid chromatography (UFLC), as previously described by Pereira et al. (2013) [17], through a UFLC system, in the following operating conditions: system: Shimadzu 20 A series UFLC (Shimadzu Corporation, Kyoto, Japan); column: SphereClone (Phenomenex, Torrance, CA, USA) reverse phase C18 column (5 μm, 250 mm × 4.6 mm i.d.); detector: PDA Shimadzu detector; mobile phase: 3.6 mM sulphuric acid; flux: 0.8 mL/min; wavelength: 215 nm; temperature: 35 °C; software: LabSolutions, LCsolutions Version 1.25.

Alongside this, the quantification of the detected organic acids, was carried out by comparing the area of the peaks with the calibration curves obtained from commercial standards of each of the compounds, and the results were expressed in g per 100 g of dry weight (dw).

#### 2.3.2. Tocopherols

For the extraction of tocopherols, 500 mg of flower sample was used, to which was added tocol (IS) and butylated hydroxytoluene (BHT, protective antioxidant of the tocopherols). To this mixture, methanol, hexane and a concentrated NaCl solution were added. After phase separation, the upper portion was collected, evaporated in a nitrogen stream and re-dissolved in hexane, and filtered (0.22 µm disposable filters), for further analysis. The profile in tocopherols was determined following an analytical procedure previously described by Barros et al. (2013) [18], through an HPLC equipment operating in the following conditions: system: pump (Knauer, Smartline system 1000, Berlin, Germany), degasser system (Smartline manager 5000), autosampler (Jasco AS-2057, Easton, MD, USA); column: Polyamide II (250 mm × 4.6 mm i.d.) normal-phase column from YMC Waters (Dinslaken, Germany); detector: fluorescence detector (Jasco FP-2020, Easton, Maryland, MD, USA); mobile phase: hexane and ethyl acetate (70:30, *v*/*v*); flux: 1 mL/min; wavelength: excitation at 290 nm and emission at 330 nm; temperature: 35 °C; software: DataApex Clarity, Version 2.4.1.43.

The calibration curves obtained for commercial standards for each of the compounds allowed the quantification of the detected compounds, using the internal standard methodology (IS-tocol), with the results expressed in mg per 100 g of dry weight (dw).

#### 2.3.3. Betacyanins

For the chromatographic analysis of the betacyanins profile, 1.5 mL of extract solution recovered from the flower samples was obtained by ultrasound assisted extraction using distilled water as solvent in the conditions of a solid/liquid ratio of 5 g/L, time—22 min and power of 500 W, in an ultrasonic system (ultrasonic homogeniser, model CY-500, Optic Ivymen Systen, Barcelona, Spain; 20 kHz frequency) equipped with a titanium probe [16]. Afterwards, this extract was filtered through a 0.22-µm disposable syringe filter and injected onto a Dionex Ultimate 3000 HPLC system (Thermo Scientific, San Jose, CA, USA), operating under the conditions described by Roriz et al. (2018) [10]. Compounds were detected with a diode detector (DAD) at 530 nm wavelength. Chromatographic data were obtained and processed using Xcalibur software (Thermo Finnigan, San Jose, CA, USA). The detected betacyanins were characterised based on their UV-Vis and mass spectrum and quantified using the calibration curve for Gomphrenin III (y = 14,670 × −19,725, R^2^ = 0.9997). The results were expressed in mg per g of extract.

### 2.4. Evaluation of Bioactive and Colouring Properties

The extract obtained according to the description stated in Section 2.4.3. was centrifuged at 480g for 10 min and the supernatant was carefully collected and lyophilised. The colour of the obtained extract was measured as described in Section 2.3.

#### 2.4.1. Evaluation of the Anti-Haemolytic Activity

The antioxidant capacity of *A. caudatus* flower extract was assessed by the oxidative haemolysis inhibition assay (OxHLIA), following a method described by Silva de Sá et al. (2019) [19]. Briefly, an erythrocyte solution (2.8%, *v/v*; 200 µL) in phosphate-buffered saline (PBS, pH 7.4) was mixed with 400 µL of either: (i) extract solution (18.75–600 μg/mL in PBS), (ii) PBS (control), (iii) water (for complete haemolysis), or (iv) trolox (7.81–250 µg/mL PBS). After pre-incubation at 37 °C for 10 min with shaking, 200 μL of 2,2′-azobis(2-methylpropionamidine) dihydrochloride (AAPH, 160 mM in PBS, from Sigma-Aldrich) were added and the optical density was measured at 690 nm every ~10 min in a microplate reader (Bio-Tek Instruments, ELX800) until complete haemolysis. Results were expressed as IC_50_ values (μg/mL) for Δ*t* of 60 and 120 min.

#### 2.4.2. Antibacterial Activity

To access the antibacterial activity of the extract, different bacteria consisting of clinical isolates of patients from hospital units in North-eastern Portugal were used. Five Gram-negative *(Escherichia coli*, *Klebsiella pneumoniae*, *Morganella morganii*, *Pseudomonas aeruginosa*, and *Proteus mirabilis*) and three Gram-positive (methicillin-resistant *Staphylococcus aureus* (MRSA), *Listeria monocytogenes*, and *Enterococcus faecalis*) bacteria were tested. The microdilution method and the rapid *p*-iodonitrotetrazolium chloride (INT; Panreac Applichem, Barcelona, Spain) colorimetric assay were performed following the procedures described by Pires et al. (2018) [20] with some modifications. For each inoculum, one positive control was prepared with bacteria and Mueller–Hinton broth (MHB; Biolab, Budapest, Hungary). A solution prepared with MHB or tryptic soy broth (TSB; Biolab, Budapest, Hungary), one with the extract only, and the last one with medium and antibiotic (vancomycin, imipenem, or ampicillin), were used as negative controls. Minimum inhibitory concentrations (MICs, mg/mL), which represent the lowest extract concentration that inhibits the visible bacterial growth, and minimum bactericidal concentrations (MBCs, mg/mL), corresponding to the lowest extract concentration required to kill a particular bacterium, were the units in which the results were expressed.

#### 2.4.3. Cytotoxicity to Normal Cells

The extract was tested for potential toxicity to normal cells, namely a porcine liver primary culture (PLP2), through the sulforhodamine B assay, as described by Guimarães et al. (2013) [21]. The extract was tested in a range of concentrations from 400 to 1.56 μg/mL, allowing the determination of the extract concentration providing 50% of cell growth inhibition (GI_50_, µg/mL). Ellipticine was used as a positive control.

### 2.5. Optimisation of the Betalains Extraction Using Response Surface Methodology

#### 2.5.1. Experimental Design for Extraction Process Optimisation

A five-level central composite design (CCD) coupled with RSM was implemented to optimise the extraction of betacyanins from *A. caudatus* flowers. The coded and real values of the independent variables *X*_1_ (time: *t*, min) and *X*_2_ (ultrasonic power: *p*, W) are presented in Supplementary Material Table S1. These variables and their range of values were selected based on a previous study of Rocha et al. [17]. The ultrasonic power covered the minimum and maximum values allowed by the ultrasonic equipment, while times greater than 45 min are not feasible due to possible degradation phenomena. Design-Expert software, Version 11 (Stat-Ease, Inc., Minneapolis, MN, USA) was used to generate the 18 experimental points of the design, which included 4 factorial points, 4 axial or star points replicated two times, and 6 points replicated at the centre of the experimental domain. The experimental runs were randomised to minimise the effects of unexpected variability.

#### 2.5.2. Ultrasound-Assisted Extraction

The UAE was performed using the ultrasonic system referred to above. A known weight (~2.5 g) of flower sample was placed in a beaker with 50 mL of distilled water and processed according to the experimental design, where different levels of *t* (1–45 min) and *P* (5–500 W) were combined. The solid/liquid ratio was kept at 50 g/L, as well as the temperature (25–35 °C; a water bath was used to avoid increasing the temperature). After extraction, the mixtures were centrifuged at 480 *g* for 10 min and the supernatants were carefully collected. An aliquot of each supernatant was used to determine the extraction yield (extract weight, %, *w*/*w*) and the remainder was used for quantification of betalains (mg/g plant material), as described in Section 2.4.3.

#### 2.5.3. Extraction Process Modelling and Statistical Analysis

The response variables *Y*_1_ (extraction yield), *Y*_2_ (amaranthine content), *Y*_3_ (isoamaranthine content), and *Y*_4_ (total betacyanins content, resulting from the sum of all detected betacyanins) were used to optimise the recovery of betacyanins from *A. caudatus* flower. Fitting procedures, coefficient estimates, and statistical analysis of the polynomial model equations were performed using Design-Expert software as previously described [22].

### 2.6. Statistical Analysis

The experiments were carried out in triplicate and the results were expressed as the mean ± standard deviation (except for antibacterial activity). For colour and antioxidant activity analyses, the SPSS Statistics Software (IBM SPSS Statistics for Windows, Version 22.0. Armonk, NY, USA: IBM Corp.) was used to assess significant differences among two samples by applying a two-tailed paired Student’s *t*-test (significance of 0.001).

## 3. Results and Discussion

### 3.1. Colour Parameters

The results of the colour parameters L* (lightness), a* (redness), and b* (yellowness) measured in the *A. caudatus* flower sample and in its extract are presented in Table 1, as well as the results of their conversion to RGB values that visually illustrate the colour shades. It was possible to observe a significant difference between the two samples. The extract presented a lower L* value and a higher a* value, thus showing a darker and reddish colour shade. In addition, the parameter b* indicated that the flower sample was more yellow than the extract. Overall, the colour was intensified after extraction, as expected, due to a concentration of betacyanin pigments, resulting in an intense purple colour. Thus, statistically significant differences in the colour parameters of the flower and the extract, can be verified (*p*-value < 0.001).

### 3.2. Chemical Profile

Although *A. caudatus* has been the subject of previous studies, the seeds have been the most studied part of this plant. Table 2 presents the *A. caudatus* flowers composition in organic acids and tocopherols. Oxalic acid (2.48 mg/100 g dw) was identified as the major organic acid, followed by shikimic acid (0.170 mg/100 g dw). Traces of fumaric acid were also detected. Although there are no previous studies describing the organic acid profile of the studied flowers, oxalates have been described in this plant [23]. Regarding tocopherols, three of the four isoforms were detected in the studied *A. caudatus* flowers. β-Tocopherol was the most abundant (0.884 mg/100 g dw), followed by the δ- (0.60 mg/100 g dw) and α (0.47 mg/100g dw) vitamers. According to the literature, there is no previous data on the tocopherols composition of *A. caudatus* flower, but there are for the seeds. Bruni et al. [24,25] reported δ (2.17–4.88 mg/100 g), β (1.95–4.39 mg/100 g) and α (1.25–3.48 mg/100 g) tocopherols as the major vitamers in *A. caudatus* seeds, and γ-tocopherol (0.06–0.22 mg/100 g) in minor concentration. The levels of tocopherols reported for the seeds are higher than those quantified in this study for flowers, as the seeds are generally richer in lipophilic constituents. The chemical characterisation herein described contributes to the knowledge of *A. caudatus* flowers in terms of organic acids and tocopherols composition, since, to the best of the authors’ knowledge, this is the first report describing these molecules in this plant matrix.

Table 3 shows the retention times, wavelengths of maximum absorption in the visible region, mass spectral data, tentative identification, and quantification of the betacyanins detected in *A. caudatus* flower extract. Four compounds were tentatively identified, namely amaranthine ([H]^+^ at m/z 727), isoamaranthine ([H]^+^ at m/z 727), betanin ([H]^+^ at m/z 551), and isobetanin ([H]^+^ at m/z 551), based on data of previous studies [26,27] made with plants of the same species. As far as the authors know, this is the first study reporting the betacyanins composition of *A. caudatus* flower (small flowers that make up the inflorescences) extracts.

Regarding the quantification of the compounds, it was possible to observe that peaks 1 and 2, amaranthine and isoamaranthine, respectively, represented together 98.58% of the total amount of betacyanins identified, with amounts of 171 ± 1 and 38 ± 1 mg/g of extract, respectively. Due to the low representative quantities of peaks 3 and 4, betanin and isobetanin, respectively, were not taken into consideration for the optimisation studies performed by UAE.

### 3.3. Bioactive Properties

The antihaemolytic activity of the *A. caudatus* flower extract was tested by the OxHLIA assay. According to the obtained results (Table 4), extract concentrations of 29 μg/mL and 111 μg/mL were required to protect half the erythrocyte population from the oxidative haemolytic induced by AAPH, a temperature-dependent free radical initiator, for Δ*t* of 60 and 120 min, respectively. Trolox, the positive control used, was more effective in protecting the erythrocyte membranes than the flower extract, with IC_50_ values of 16.6 μg/mL and 44 μg/mL for Δ*t* of 60 and 120 min, respectively. However, it should be noted that the trolox is a pure antioxidant compound, whereas the tested extract is a complex mixture of different constituents, some of which have no activity. In addition, it was also found that the antioxidants present in the flower extract were more effective for a period of just 60 min than for 120 min. Antioxidants, such as betacyanins and tocopherols, probably acted and were depleted at an earlier stage of the in vitro assay. In a previous study, Jo et al. [22] evaluated the radical scavenging activity of *A. caudatus* flower extract obtained by hot water extraction and reported it as a promising source of antioxidants. However, it is difficult to compare these results with those of the present study due to the different mechanisms of action of the performed antioxidant assays.

Regarding the antibacterial activity (Table 4), the flower extract showed activity against most of the tested microorganisms, with the exception of *L. monocytogenes* that was not inhibited at the maximum tested concentration of 20 mg/mL. In general, the tested Gram-negative bacteria showed higher susceptibility to the flower extract. In some cases, the MIC values shown by the extract are lower. For instance, the extract concentrations needed to inhibit *K. pneumoniae* and *M. morganii* were 5 and 10 mg/mL, respectively, while the concentrations of ampicillin needed to inhibit these microorganisms were 10 mg/mL and 20 mg/mL, respectively. The obtaining of natural extracts with a similar/higher antibacterial potential than the commonly used antibiotics is of high relevance, given the growing inefficacy of antibiotics against bacterial strains.

For this species, regarding the bioactivities, the data in the literature refer mostly to the seeds of this plant, which are the plant parts that have aroused the most interest so far. In a review study conducted by Martinez-Lopez et al. [23], the authors state that the seeds contain a large number of bioactivities such as anthelmintic, antinociceptive, antipyretic, anticancer, antiallergenic, antidiabetic, stimulation of the immune system, cardioprotective, hepatoprotective and antibacterial activities. According to Maiyo et al. [28], *A. caudatus* fresh leaf extracts where active against *E. coli*, *Salmonella typhi*, *Prooteus mirabilis*, *Staphylococcus aureus*, and *Bacillus* sp.

Regarding the toxicity for normal cells PLP2, the extract revealed no toxicity at a maximum of 400 μg/mL, allowing the attainment of an indication of its safety. Therefore, other in vitro and in vivo assays are needed to validate these results.

Additionally, regarding the described bioactivities, it should be noted that these results refer to the seeds of this species, and for that reason different results are obtained compared to the present work. These differences can be explained due to differences in the chemical composition of the different parts of the plant, since in the present study, the main focus is the recovery of colouring compounds (betacyanins) and the analysis of their ability to promote biological properties as an extra benefit.

### 3.4. Optimisation of the Extraction Process

Betacyanins can be recovered from plant materials using conventional solid–liquid extraction methods or novel extraction techniques as alternatives to the conventional ones to increase the betalains’ yield in a more efficient and sustainable way. In recent years, several studies have been carried out to optimise the extraction of these pigments from various plant matrices using different methodologies. In this study, the suitability of UAE for extracting betacyanins from *A. caudatus* was investigated and optimised. This time-saving method has already been successfully applied by the authors to recover betacyanins from globe amaranth (*Gomphrena globosa* L.) flowers [12,16]. It allows the improvement of the extraction yield due to the acoustic cavitation effect that enhances mass transfer phenomena [29,30]. However, the energy applied to promote the plant cell disruption can affect the molecules integrity if applied at high intensity, as well as the other independent variables used in the process intensification. The most relevant ones should be combined in experimental RSM designs, in order to access interactive effects and to optimise extraction process with a low number of executions.

#### 3.4.1. Experimental Data for Process Optimisation

As presented in Table 5, the extraction yield (extract weight) achieved with the 18 runs of the CCD design was quite low and ranged from 1.31 to 2.27% (*w*/*w*). In turn, as discussed above, amaranthine and isoamaranthine were the major betacyanins identified in *A. caudatus* flowers. The amaranthine levels ranged from 48.87 to 55.70 mg/g plant material, while the isoamaranthine levels ranged from 16.52 to 19.56 mg/g plant material. In both cases, the lower and higher contents were obtained with the runs 1 and 4 (which combined the independent variables at medium–low (−1) and medium–high (+1) levels) and 2 and 3 (which combined the independent variables at medium–high (+1) and medium–low (−1) levels), respectively. For the first case, this observation indicates that the combined variables’ intensity may not be sufficient to promote the extraction, due to the short time and low ultrasonic power involved, or that, if both variables are applied at a higher intensity, they may lead to pigment degradation. According to the results of runs 2 and 3, it is preferable to use a longer processing time combined with a lower ultrasonic power, or a shorter sonication time with a higher ultrasonic power to promote extraction without causing degradation. The experimental runs achieved with the fourth response variable (total betacyanins) showed the same trend, as it resulted from the sum of all betacyanins quantified in the aqueous flower extract, where amaranthine (~70%) and isoamaranthine (~24%) were major compounds. Indeed, as betanin and isobetanin were detected at lower concentration (~6% of the total content), the optimisation was performed only for amaranthine and isoamaranthine in an individual way (Table 5).

#### 3.4.2. Models Fitting and Statistical Verification

The response values in Table 5 were fitted to a polynomial regression model using the Design-Expert software, but just the significant parameters (assessed at a 95% confidence level) were used in the construction of the predictive models. The results of analysis of variance and regression analyses are presented in Supplementary Material Table S2, while the resulting polynomial models are shown in Equations (1)–(4).

(1) $$Y_{\left(Yield\right)}=1.73+0.186t+0.236P\;[R^{2}\;=\;0.8638;\;{R^{2}}_{\mathrm{adj}}\;=\;0.8457]$$

(2) $$Y_{\left(Amaranthine\right)}=52.08-3.35tP\;[R^{2}\;=\;0.8178;\;{R^{2}}_{\mathrm{adj}}\;=\;0.7788]$$

(3) $$Y_{\left(Isoamaranthine\right)}=18.05-1.18tP\;[R^{2}\;=\;0.7526;\;{R^{2}}_{\mathrm{adj}}\;=\;0.7000]$$

(4) $$Y_{\left(Total\;betacyanins\right)}=75.57-4.70tP\;[R^{2}\;=\;0.7881;\;{R^{2}}_{\mathrm{adj}}\;=\;0.7439]$$

In the mathematical models developed for each response variable, the coefficients illustrate the effect of the independent variables and/or their interaction. The parametric values represent the expected effect on the response when changing one factor value. Therefore, the higher these values, the more significant the weight of the respective variable will be. In addition, the negative sign represents an antagonistic interaction between variables [30]. In each model equation, the intercept corresponds to the overall average response of the 18 runs of the CCD design (Table 5). As discussed above, these values are low for the extraction yield (extract weight) and particularly higher for amaranthine and total betacyanin contents.

The four mathematical models had a non-significant lack-of-fit (*p* > 0.05) and an adequate precision > 15, which indicates that the model equations adequately describe the effects of the variables *t* and *P* on *Y*_1_–*Y*_3_ [31]. The coefficients R^2^ and R^2^_adj_ were higher than 0.75 and 0.70 for all models, respectively (Equations (1)–(4)), showing that the variability of each response can be explained by the extraction process variables. Value > 15.4 were obtained for adequate precision (Supplementary Material Table S2). Therefore, the constructed model equations were statistically validated and used in the following steps to predict the optimal UAE conditions.

Based on Equation (1), it can be observed that the extraction yield is significantly affected by both independent variables through linear effects, while, for betacyanins, Equations (2)–(4) illustrate that the extraction process is affected by negative interactive effects between the variables. These results support the use of RSM as an optimisation tool, since the one-factor-at-a-time approaches do not evaluate interactive effects.

#### 3.4.3. Effect of the Independent Variables on the Target Responses

**Figure 1:**
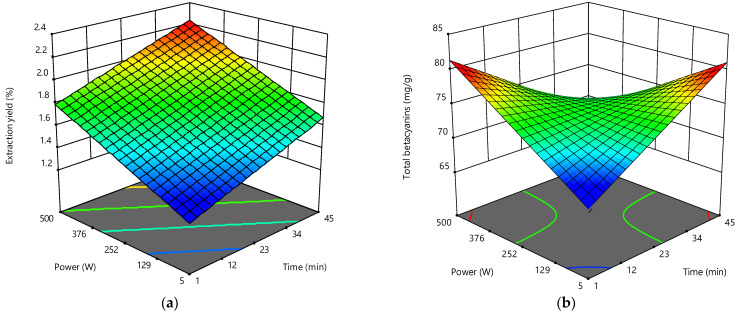
Response surface graph illustrating the binary effects of the two independent variables on (**a**) extraction yield (%, *w*/*w*) and (**b**) total betacyanins content (mg/g plant material) obtained from *A. caudatus*.

The 3D response surface graphs generated to illustrate the effect of the two independent variables on the extraction yield and total betacyanin contents are presented in Figure 1. The extraction yield was positively affected by the two independent variables involved in the extraction process, mainly by ultrasonic power. As shown in Figure 1, the increase in extraction time and ultrasonic power led to a linear increase in response values. On the other hand, sonication of the flower sample at low power for short periods of time was not sufficient to promote the recovery of betacyanins, while processing at high ultrasonic power over longer periods appeared to have caused pigment degradation. The red coloured response surface areas show two possible response optimums, corresponding to the use of short processing times at high ultrasonic power, and vice versa. Thus, based on the 3D representations, it is possible to conclude that the greater extract weight obtained with the combination of extreme processing conditions does not translate to a greater extraction of betacyanin pigments.

According to previous reports, the betacyanin stability can be affected by several factors such as temperature, pH, light intensity, and presence of oxygen and enzymes, among others [32]. Therefore, the intensification factors to be used in extraction processes must be applied in sufficient intensity to promote the pigments’ recovery, but that does not harm its integrity. In fact, when exposed to extreme processing conditions, betacyanins can undergo isomerisation, deglycosylation, or decarboxylation depending on their structural features [33].

#### 3.4.4. Optimal Extraction Conditions

The optimal extraction conditions predicted with the theoretical model Equations (1)–(4) for each response variable are presented in Supplementary Material Table S3 and can be summarised as follows: (i) 2.14 ± 0.05% extract weight (*w*/*w*) is obtained by processing the plant material at 474 W for 38.5 min; (ii) 55.2 ± 0.6 mg/g pant material of amaranthine are obtained by processing at 443 W for 4.2 min; (iii) 19.4 ± 0.3 mg/g pant material of isoamaranthine are obtained by processing at 473 W for 6.49 min; and (iv) 79.2 ± 0.9 mg/g pant material of total betacyanins are obtained by processing at 454 W for 4.8 min. Overall, the recovery of betacyanins required a shorter extraction time than the extraction yield, while high ultrasonic powers were involved in all cases. In this study, the optimal extraction conditions that maximise both the extraction yield and the total betacyanin content as much as possible were also determined (Supplementary Material Table S3), since it is important to achieve a high extract weight with high levels of colouring compounds for further application in the industrial sector as natural colorants. Based on this second optimisation, it was concluded that 13.3 min sonication at 500 W were the optimal UAE conditions to simultaneously maximise the response variables (1.92 ± 0.05% extract and 77.6 ± 0.7 mg/g of total betacyanins).

A previous study performed by the authors in globe amaranth flowers [16], 22 min sonication at 500 W (20 kHz) was described as the UAE conditions that maximise the aqueous extraction of betacyanins. The longer processing time can be justified by the intrinsic nature of the plant material and by the different betacyanin compounds. One other author [13] described 37 min as the optimal MAE time for the pigments’ recovery from *Bougainvillea glabra* Choisy flowers, when applying a lower ultrasonic power of 88 W (20 kHz) and water at 55 °C. In turn, Melgar et al., [34] and Ahmed et al. [35] reported time-saving UAE processes for extraction of betalains from prickly pear (*Opuntia engelmannii* Salm-Dyck ex Engelm.) peel (2.5 min processing at 2 kHz and 200 rpm, using 34.6% methanol at pH 7) and red amaranth (*Amaranthus cruentus* L.) leafy part (5 min sonication at 35 kHz, using water at 70 °C).

## 4. Conclusions

The chromatographic analyses allowed characterisation of the profile in organic acids, tocopherols, and betacyanins of the underexploited *A. caudatus* flowers. The aqueous extract display antihemolytic activity and antibacterial effects against foodborne bacteria. Furthermore, it has no hepatotoxicity to PLP2 cells at the tested concentrations, which represents a positive clue regarding safety issues. Moreover, given the high amounts and colouring capacity of betacyanins, the UAE of these pigments was optimised by RSM in order to obtain a betacyanin-rich purple extract. The UAE process was affected by both independent variables, extraction time and ultrasonic power. The predictive models were successfully fitted to the experimental data and validated based on different statistical criteria. With 13.3 min sonication at 500 W, it was possible to reach an overall maximum of 1.92% of extract weight (*w*/*w*) and 77.6 mg/g of total betacyanins. This betacyanin-rich extract can be highlighted as a promising natural colorant for application mainly in the food sector as an alternative to the controversial artificial food colorants since this ingredient can also act as a preservative and health promoter due to its antioxidant and antibacterial properties.

## Figures and Tables

**Table 1:** Colour parameters of the lyophilised flower sample and its extract, according to the CIELAB colour space and the RGB scale.

	CIELAB			RGB			Colour
	L*	a*	b*	R	G	B	
Flower sample	56.40 ± 0.06	17.78 ± 0.01	2.61 ± 0.01	155	123	128	
Flower extract	21.76 ± 0.02	31.45 ± 0.06	1.95 ± 0.03	82	34	53	
*p*-value	<0.001	<0.001	<0.001	<0.001	<0.001	<0.001	

L* (lightness), a* (greenness–redness), and b* (blueness–yellowness).

**Table 2:** Tocopherols and organic acids composition of *A. caudatus* flowers.

Organic Acids	Content (g/100 g dw)
Oxalic acid	2.48 ± 0.05
Shikimic acid	0.170 ± 0.003
Fumaric acid	tr
Total	2.65 ± 0.03
Tocopherols	Content (mg/100 g dw)
α-Tocopherol	0.47 ± 0.01
β-Tocopherol	0.884 ± 0.003
γ-Tocopherol	nd
δ-Tocopherol	0.60 ± 0.06
Total	1.95 ± 0.06

tr: traces; nd: not detected.

**Table 3:** Betacyanins of *A. caudatus* flower extract chromatographic identification (retention time (Rt)), wavelengths of maximum absorption in the visible region (λ_max_), and mass spectral data (MS), and quantification (mg/g extract).

Peak	Rt (min)	λ_max_ (nm)	[H]^+^ (m/z)	MS^2^	Tentative Identification	Quantification mg/g Extract
1	18.68	536	727	551(100), 389(40)	Amaranthine	171 ± 1
2	20.02	536	727	551(100), 389(27)	Isoamaranthine	38 ± 1
3	22.04	536	551	389(100)	Betanin	1.6 ± 0.1
4	23.23	536	551	389(100)	Isobetanin	1.3 ± 0.1
					Total	212 ± 1

**Table 4:** Antihaemolytic and antibacterial activities of the *A. caudatus* flower extract and positive controls.

	Flower Extract		Positive Controls					
Antihaemolytic activity (IC_50_, µg/mL)			Trolox					
OxHLIA, Δ*t* 60 min	29 ± 1		19.6 ± 0.8					
OxHLIA, Δ*t* 120 min	111 ± 5		41 ± 1					
Antibacterial activity (MIC and MBC, mg/mL)			Ampicillin (20 mg/mL)		Imipenem (1 mg/mL)		Vancomycin (1 mg/mL)	
	MIC	MBC	MIC	MBC	MIC	MBC	MIC	MBC
Gram-negative bacteria								
*Escherichia coli*	10	>20	<0.15	<0.15	<0.0078	<0.0078	nt	nt
*Klebsiella pneumoniae*	5	>20	10	20	<0.0078	<0.0078	nt	nt
*Morganella morganii*	5	>20	20	>20	<0.0078	<0.0078	nt	nt
*Proteus mirabilis*	20	>20	<015	<0.15	<0.0078	<0.0078	nt	nt
*Pseudomonas aeruginosa*	20	>20	>20	>20	0.5	1	nt	nt
Gram-positive bacteria								
*Enterococcus faecalis*	5	>20	<0.15	<0.15	nt	nt	<0.0078	<0.0078
*Listeria monocytogenes*	>20	>20	<0.15	<0.15	<0.0078	<0.0078	nt	nt
MRSA	5	>20	<0.15	<0.15	nt	nt	0.25	0.5

MRSA—methicillin Resistant *Staphylococcus aureus*; for antihemolytic activity, statistical differences (*p* < 0.001) between samples were found when applying a Student’s *t*-test. MIC: minimum inhibitory concentration; MBC: minimum bactericidal concentration; nt: not tested.

**Table 5:** Experimental results for the betacyanins content obtained with the 18 runs of the central composite design (CCD) design.

Runs	Experimental Domain		Experimental Responses			
	Time (min)	Power (W)	Extraction Yield (%, *w*/*w*)	Amaranthine (mg/g Plant Material)	Isoamaranthine (mg/g Plant Material)	Total Betacyanins * (mg/g Plant Material)
1	−1 (4.5)	−1 (44.4)	1.31	48.87	16.52	69.55
2	+1 (41.5)	−1 (44.4)	1.54	55.46	18.85	78.94
3	−1 (4.5)	+1 (460.6)	1.91	55.70	19.56	79.90
4	+1 (41.5)	+1 (460.6)	2.20	48.90	17.16	70.48
5	−1.19 (1)	0 (252.5)	1.42	52.26	17.84	74.36
6	−1.19 (1)	0 (252.5)	1.45	52.26	18.64	75.41
7	+1.19 (45)	0 (252.5)	1.70	51.53	17.76	73.77
8	+1.19 (45)	0 (252.5)	2.03	52.39	18.34	75.32
9	0 (23)	−1.19 (5)	1.60	52.40	17.87	74.83
10	0 (23)	−1.19 (5)	1.44	52.41	18.11	75.03
11	0 (23)	+1.19 (500)	1.85	51.85	17.76	73.84
12	0 (23)	+1.19 (500)	2.27	51.85	18.45	74.79
13	0 (23)	0 (252.5)	1.74	51.87	17.86	74.00
14	0 (23)	0 (252.5)	1.71	53.34	18.39	76.31
15	0 (23)	0 (252.5)	1.85	50.67	17.87	73.06
16	0 (23)	0 (252.5)	1.80	52.34	18.16	75.22
17	0 (23)	0 (252.5)	1.75	50.06	17.29	71.23
18	0 (23)	0 (252.5)	1.63	53.32	18.46	76.23

* Sum of the amaranthine, isoamaranthine, betanin, and isobetanin contents.

## Data Availability

Data is contained within the Supplementary Material.

## Supplementary Materials

The following are available online at https://www.mdpi.com/article/10.3390/foods10040779/s1, Table S1: Natural and coded values of the independent variables used in the five-level CCD design implemented to optimise the extraction process of betacyanins from Amaranthus using RSM. Table S2: Parametric coefficients and statistical information of the fitting procedure of the models. Parametric superscripted 1 and 2 stands for the variables time and ultrasonic power, respectively. Table S3: Optimal extraction conditions in natural values that maximise the response values.
