# Supplementary material for: Chemical and Bioactive Features of Amaranthus caudatus L. Flowers and Optimized Ultrasound-Assisted Extraction of Betalains
Source: Foods. 2021 Apr 5;10(4):779. doi: 10.3390/foods10040779 (PMC8067032; doi:10.3390/foods10040779)
Supplement: Supplementary file 1 [file foods-10-00779-s001.pdf]

foods MDPI

Supplementary Material

Table S1. Selected and coded values of the independent variables used in the five-level CCD design implemented to optimize the extraction process of betacyanins from *Amaranthus* using RSM.

Coded values	Selected values	
	Time (min)	Ultrasonic power (W)
−1.189	1	5
−1	4.5	44.4
0	23	252.5
+1	41.5	460.6
+1.189	45	500

Table S2. Parametric coefficients and statistical information of the fitting procedure of the models. Parametric superscripted 1 and 2 stands for the variables time and ultrasonic power, respectively.

		Extraction yield	Amaranthine	Isoamaranthine	Total betacyanins
Intercept	b_0	1.73 ± 0.02	52.1 ± 0.2	18.05 ± 0.09	74.6 ± 0.3
Linear effect	b_1	0.19 ± 0.03	ns	ns	ns
	b_2	0.24 ± 0.03	ns	ns	ns
Quadratic effect	b_{11}	ns	ns	ns	ns
	b_{22}	ns	ns	ns	ns
Interactive effect	b_{12}	ns	−3.4 ± 0.4	−1.2 ± 0.2	−4.7 ± 0.7
**Statistical data**					
Model F-value		47.58	20.95	14.19	17.46
Lack of Fit		ns	ns	ns	ns
R^2		0.8638	0.8178	0.7526	0.7891
R^2_{adj}		0.8457	0.7788	0.7000	0.7439
Adequate Precision		21.60	17.32	15.41	15.63
C.V. (%)		5.52	1.63	2.06	1.74

R^2: coefficient of determination; R^2_{ajd}: adjusted coefficient of determination; C.V.: coefficient of variation; ns: not significant.

Table S3. Optimal extraction conditions that maximize the response values.

	Optimal UAE conditions		Response optimum
	Time (min)	Power (W)	
For each response variable			
Extraction yield	38.5	474.3	2.14 ± 0.05 % (*w/w*)
Amaranthine	4.2	443.0	55.2 ± 0.6 mg/g plat material
Isoamaranthine	6.49	473.3	19.4 ± 0.3 mg/g plat material
Total betacyanins	4.8	454.2	79.2 ± 0.9 mg/g plat material

Considering all response variables			
Extraction yield			1.92 ± 0.05 % (w/w)
Amaranthine	13.3	500	54.1 ± 0.5 mg/g plat material
Isoamaranthine			19.0 ± 0.2 mg/g plat material
Total betacyanins			77.6 ± 0.7 mg/g plat material
